# Comprehensive Analysis of Titanium Oxide Nanoparticle Size and Surface Properties on Neuronal PC-12 Cells: Unraveling Cytotoxicity, Dopaminergic Gene Expression, and Acetylcholinesterase Inhibition

**DOI:** 10.3390/jox13040043

**Published:** 2023-11-07

**Authors:** Jitendra Kumar Suthar, Balaji Rakesh, Anuradha Vaidya, Selvan Ravindran

**Affiliations:** 1 Symbiosis School of Biological Sciences, Faculty of Medical and Health Sciences, Symbiosis International (Deemed) University, Pune 412115, India; jitendra.suthar@ssbs.edu.in; 2 Symbiosis Institute of Technology, Symbiosis International (Deemed) University, Pune 412115, India; balaji.rakesh@sitpune.edu.in; 3 Symbiosis Centre for Stem Cell Research, Symbiosis School of Biological Sciences, Symbiosis International (Deemed) University, Pune 412115, India; director@ssbs.edu.in

**Keywords:** neurotoxicity, titanium oxide, nanoparticles, oxidative stress, PC-12 cells

## Abstract

Titanium oxide nanoparticles can penetrate the blood–brain barrier, infiltrate the central nervous system, and induce neurotoxicity. One of the most often utilized nanoparticles has been investigated for their neurotoxicity in many studies. Nonetheless, there remains an unexplored aspect regarding the comparative analysis of particles varying in size and nanoparticles of identical dimensions, both with and devoid of surface coating. In the current study, we synthesized two differently sized nanoparticles, TiO_2_-10 (10 nm) and TiO_2_-22 (22 nm), and nanoparticles of the same size but with a polyvinylpyrrolidone surface coating (TiO_2_-PVP, 22 nm) and studied their toxic effects on neural PC-12 cells. The results highlighted significant dose- and time-dependent cytotoxicity at concentrations ≥10 μg/mL. The exposure of TiO_2_ nanoparticles significantly elevated reactive oxygen and nitrogen species levels, IL-6 and TNF-α levels, altered the mitochondrial membrane potential, and enhanced apoptosis-related caspase-3 activity, irrespective of size and surface coating. The interaction of the nanoparticles with acetylcholinesterase enzyme activity was also investigated, and the results revealed a dose-dependent suppression of enzymatic activity. However, the gene expression studies indicated no effect on the expression of all six genes associated with the dopaminergic system upon exposure to 10 μg/mL for any nanoparticle. The results demonstrated no significant difference between the outcomes of TiO_2_-10 and TiO_2_-22 NPs. However, the polyvinylpyrrolidone surface coating was able to attenuate the neurotoxic effects. These findings suggest that as the TiO_2_ nanoparticles get smaller (towards 0 nm), they might promote apoptosis and inflammatory reactions in neural cells via oxidative stress, irrespective of their size.

## 1. Introduction

As advances in nanoscience and nanotechnology accelerate, more attention is being focused on addressing the human health risks and potential toxicities associated with the widespread use of nanoparticles (NPs). Titanium oxide nanoparticles (TiO_2_ NPs) are among the top five engineered NPs used in consumer products, with a broad spectrum of applications in plastics, paints, energy storage, and generating modules such as batteries and solar panels [1], posing a risk of an indirect exposure of these NPs to humans.

However, the application of TiO_2_ NPs in the food and medical industries poses a significantly greater risk than their industrial application [2]. In the food industry, TiO_2_ NPs are used for packaging and additives [3]; in the medical sector, they are used in products like dental and orthopedic implants, wound dressings, diagnostic imaging, stents, and vascular grafts [4,5]. Due to its high reactivity, titanium (Ti) rapidly develops a protective TiO_2_ layer when exposed to air or fluids (passivation), which shields the metal. However, the integrity of this layer can deteriorate when subjected to tribocorrosion, which occurs when TiO_2_ NPs come into contact with surfaces or materials and experience both mechanical wear and electrochemical corrosion [6,7]. As a result, Ti particles or ions may be released into the cellular environment, and thus, it raises concerns about their potential systemic toxic effects.

TiO_2_ has been classified as Group 2B by the International Agency for Research on Cancer and the National Institute for Occupational Safety and Health, suggesting its probable carcinogenicity in humans [8]. Multiple studies have demonstrated evidence of TiO_2_ NPs’ adverse effects, such as hepatotoxicity [9,10], nephrotoxicity [11], respiratory [12], and cardiovascular toxicity [13]. However, considering their relatively small size and significant surface area, NPs have unique characteristics that allow them to translocate to the central nervous system (CNS) via blood circulation. Significant routes for this translocation involve the passage through the blood–brain barrier (BBB) [14], the olfactory nerve [15], or via the placental barrier to the fetal brain [16]. The metal accumulation in the brain, particularly redox metals, such as titanium, sodium, magnesium, iron, and zinc, may cause amplified oxidative stress (with the generation of excess hydroxyl and superoxide radicals), which may be linked with severe neuronal damage in both normal aging and neurodegenerative disorders (NDs) [17,18].

It is well known that increasing the surface area speeds up the dissolving processes. Smaller particle sizes paired with faster dissolution rates increase their absorption across membranes, culminating in accumulation within tissues and organs following oral administration [19,20]. However, TiO_2_ has a prolonged dissolving rate compared to other metallic NPs [21]. Due to this lower dissolution rate, the toxic effects induced via TiO_2_ NPs are primarily because of the particle’s characteristics and not the metallic ions released from them. The NPs can gain entry into the body via multiple routes, such as oral/ingestion [22], dermal/topical [23], inhalational, and injection [24,25]. Aggregation of TiO_2_ NPs occurs in near-neutral pH solutions but may dissociate in acidic solutions [20]. These NPs follow first-order dissolution kinetics, suggesting low solubility and extended half-life, indicating their ability to exist in an unchanged form for extended durations in the body, inducing acute and chronic health hazards [21]. Therefore, even if there is no release of Ti ions from TiO_2_ NPs at any concentration, as seen in the case of zinc, copper, or silver NPs, constant vigilance and in-depth analysis of specific NP characteristics are essential to ensure comprehensive safety precautions.

The existing literature on TiO_2_ NPs’ neurotoxicity yields mixed results, with few studies demonstrating toxic impacts and others demonstrating contrary results. Numerous studies have investigated the impact of particle size on neurotoxicity; however, these studies have frequently compared particles with a low likelihood of crossing the BBB or to the bulk form of identical particles. Such comparative studies do not accurately reflect the true impact of particle size on neurotoxicity, as a more pertinent focus should be on particles within the size range that have a higher probability of reaching the CNS. Smaller NPs with a size of approximately ≤100 nm have higher chances of penetrating through the BBB via endocytosis through the cells or through the transmembrane or the paracellular pathway and enter the CNS [26,27,28,29,30].

Therefore, the current research aimed to study the neurotoxicity of TiO_2_ NPs to assess the impact of size and surface coating on the neurotoxicity induced. For this, TiO_2_ NPs of two different sizes and particles of similar size, with and without surface coating, were synthesized to address this issue. This comprehensive approach allows for evaluating the combined impact of particle size and surface coating on the toxicity induced. The polymer polyvinylpyrrolidone (PVP) was selected to assess the impact of coating on neurotoxicity to ensure comparability with other research endeavors. PC-12 cells were selected as the preferred model for the current research, as they are particularly suitable for examining the neurotoxic effects due to their capacity to differentiate into neuron-like cells [31]. In addition to assessing the relationship between NPs and NDs, the current study also explored the effect of TiO_2_ NPs on the expression of genes linked with the dopaminergic system in PC-12 cells. The genes tyrosine hydroxylase (Th), monoamine oxidase A (MaoA), and catechol-o-methyltransferase (Comt) were selected for their association with dopamine metabolism [32]. The genes α-synuclein (Snca), parkin (Park2), and G protein-coupled receptor 37 (Gpr37) were selected to explore the connection between the onset of neurodegeneration and the neurotoxicity of TiO_2_ NPs [33].

Apart from neurotoxicity and the possible association with NDs it triggers, the significance of TiO_2_ NPs also lies in their interaction with crucial enzymes, notably acetylcholinesterase (AChE). This interaction is of significant importance due to the pivotal role that AChE plays in essential neurological processes, including neurotransmission, cognitive functions, and memory [34]. NPs might attach to AChE after intake and modify the enzyme’s activity. This study further investigated the interaction of TiO_2_ NPs with AChE enzyme activity.

## 2. Materials and Methods

### 2.1. Nanoparticle Synthesis

TiO_2_ NPs were synthesized using the sol–gel method with ethanol as a solvent, titanium tetrachloride (TiCl_4_ ≥ 99.0% purity, Sigma-Aldrich, Burlington, MA, USA) as a precursor, and ammonia (25%, SRL Chemicals, Mumbai, India) as a stabilizer. The Ti precursor was mixed with double distilled water in an ice bath under constant stirring. To this precursor solution, drop-wise addition of the ethanol (99% purity, Merck, Darmstadt, Germany) was performed, and further ammonia was added under constant stirring till the formation of a white precipitate with a semi-solid consistency was obtained. The precipitate was washed 5–6 times using double distilled water and dried in a hot oven at 80 °C. Upon complete moisture removal, it was finely grounded and annealed at 400 °C and 500 °C.

PVP-coated TiO_2_ NPs were synthesized hydrothermally by mixing titanium tetra-isopropoxide (97% purity, Sigma-Aldrich) (cationic precursor) and potassium nitrate (99% purity, Sigma-Aldrich) (anionic precursor) in double distilled water. PVP (0.5%) (Sigma-Aldrich) was added to this aqueous mixture under constant stirring for 2–3 h and transferred to a hydrothermal autoclave reactor at 180 °C for 1 h, which was then calcined at 500 °C (4 h) to obtain PVP-coated TiO_2_ NPs.

### 2.2. Nanoparticle Characterizations

The field-emission scanning electron microscope (FE-SEM) (FEI Nova NanoSEM 450, Hillsboro, OR, USA) was used to analyze the size and shape of the NPs that were examined. The mean diameter of the NPs was calculated using ImageJ (National Institutes of Health, New York, NY, USA) from the FE-SEM images. Further, the energy-dispersive detector (EDS) (Bruker XFlash 6I30, Billerica, MA, USA) was used for the elemental detection. The functional group determination was conducted (400–4000 cm^−1^) using the Bruker Tensor-27 FT-IR Spectrometer (KBR pellet method). The hydrodynamic diameter of the NPs was determined via dynamic light scattering (DLS) (Sympatec Nanophox) in the deionized water at 1 mg/mL for the dispersion of the NPs. The Horiba SZ-100 nanoparticle analyzer was used to measure the electrostatic potential of the particle’s shear plane in an ultrasonicated 1 mg/mL dispersion in cell culture media at room temperature.

The crystallite size of the NPs was determined via X-ray diffraction (D8 DISCOVER—Bruker) to obtain diffraction data with Cu Kα radiation (λ = 1.54056). The samples were scanned in the 20–80° range with an operating voltage of 40 kV at 40 mA.

The optical characteristics of the TiO_2_ NPs were examined using a solid-state UV–Vis spectrophotometer (Jasco, Oklahoma City, OK, USA, 200–800 nm). The link between the band gap of the metal NPs and their cellular redox potential may provide insight into why some substances are hazardous and produce oxidative stress. The NP’s optical bandgap was established through extrapolating the absorption edge of Tauc’s figure from the measured absorption spectra.

### 2.3. Culture of PC-12 Cells and Exposure of Nanoparticles

The NCCS (National Centre for Cell Science, Pune, India) provided the PC-12 cells. The cells were subsequently cultured in a T-75 flask with a complete growth culture medium (Kaighn’s Modification of F-12 Ham Nutrient Mixture comprising 5% heat-inactivated fetal bovine serum, 10% heat-inactivated horse serum, and an antibiotic solution with 10,000 U penicillin and 10 mg streptomycin) (Himedia, Mumbai, India) in a CO_2_ incubator at 37 °C (Thermo Fisher Scientific, Waltham, MA, USA) until 80–90% confluency was achieved. PC-12 cells were differentiated using nerve growth factor according to the protocol for gene expression studies [31].

A comprehensive study was conducted to mitigate the potential reduction in cell viability resulting from the gradual exhaustion of essential nutrients over an extended 96 h treatment duration. This was accomplished by carefully examining the ideal cell density (2000 − 5 × 10^4^) per well that could sustain minimal cell death, based on which a 1 × 10^4^ cell density was selected for further studies. All the synthesized NPs were sterilized through autoclaving and then dispersed via probe sonication in cell culture media.

### 2.4. Determination of Intracellular Titanium Ions

The concentration of intracellular TiO_2_ NPs was evaluated after 96 h of exposure (10 μg/mL), selected based on cell viability assays as the minimal concentration to produce cytotoxicity. The cells, post-exposure to NPs, were treated with 6 M nitric acid, subjected to PBS washes, centrifuged, dispersed in PBS (phosphate-buffered saline), and then examined using Shimadzu AA-7000’s flame atomic absorption spectrometer (FAAS, Kyoto, Japan).

### 2.5. Cell Viability Determination via the MTT and NRU Assays

PC-12 cells were cultured in a 96-well plate, incubated overnight (at 37 °C and 5% CO_2_), then exposed to varying concentrations of TiO_2_ NPs (0.1 µg/mL, 1 µg/mL, 10 µg/mL, 50 µg/mL, and 100 µg/mL) and incubated for 24 h, 48 h, 72 h, and 96 h for comparing a dose- and time-dependent impact of NPs on PC-12 cells. Furthermore, to prevent NPs from interfering with the MTT reagent, each concentration of NPs in complete growth media was treated alone simultaneously, serving as a NP blank. After exposure, the medium was substituted with 100 μL of MTT (50 μg/mL in serum-free media), incubated for 4 h in the dark, and further solubilized using DMSO (100 μL/well). The spectrophotometric measurement was recorded at 570 nm using a microplate reader. The cells with no exposure to NPs were taken as a control.

The neutral red uptake (NRU) assay was used to assess the lysosomal activity in PC-12 cells using the Neutral Red Cell Assay Kit (Himedia) [35]. A NP blank was also employed, as in the case of the MTT assay.

### 2.6. Membrane Integrity Determination via the LDH Assay

The cells were cultured in 96 wells and incubated with (0.1–100 µg/mL) TiO_2_ NPs for 24–96 h. After exposure, the supernatant from the wells was separated, and the lactate dehydrogenase (LDH) assay was performed using the relevant assay kit (LDH Cell assay, Himedia), following the manufacturer’s protocol.

### 2.7. Mitochondrial Membrane Potential (MMP)

The PC-12 cells were exposed to TiO_2_ NPs for 24–96 h. After the exposure period, JC-10 dye (50 µL/well) was added and incubated at 5% CO_2_ and 37 °C for 60 min in the dark. The fluorescence intensity was measured and used for ratio analysis. The ratio of red/green fluorescence intensity was used to determine the MMP (at λex = 490/λem = 525 nm and λex = 540/λem = 590 nm).

### 2.8. Estimation of Reactive Oxygen and Nitrogen Species

At the end of each exposure interval, the supernatant was removed, and the cells were washed with PBS. After washing, 100 µL/well of DCFDA was added and incubated for 45 min in the dark, and the fluorescence was measured using a microplate reader (Ex/Em = 485/535 nm).

The generation of nitric oxide species in response to exposure to TiO_2_ NPs was measured using the Griess reagent method [36]. At the exposure time, the supernatant from each well was collected, and the assay was performed according to the manufacturer’s procedure (Nitric Oxide Estimation Kit, Himedia). After the test, the fluorescence intensity was calculated at Ex/Em = 560/590 nm.

### 2.9. IL-6 and TNF-α via ELISA

An ELISA Kit (Invitrogen, Waltham, MA, USA) was employed to measure the concentrations of IL-6 and TNF-α. The PC-12 cells were treated with TiO_2_ NPs for 24 h, after which the cell culture supernatants were separated, and an ELISA assay was performed as per the manufacturer’s protocol.

### 2.10. Apoptosis via Caspase-3 via ELISA

Following the manufacturer’s protocol, the Caspase-3 DEVD-R110 Fluorometric Assay kit (Biotium, Fremont, CA, USA) was utilized to determine whether the caspase-3 pathway was activated via TiO_2_ NP exposure. Varied concentrations of the TiO_2_ NPs (1–100 μg/mL) were exposed to the PC-12 cells and then incubated for 24 h (at 37 °C and 5% CO_2_). After the specific time, fluorescence intensity was assessed at λex = 470 and λem = 520 nm.

### 2.11. AChE Enzyme Activity Assay

The assessment of TiO_2_ NPs (1–100 μg/mL) on AChE activity was conducted via the Ellman assay, employing the AChE Inhibitor Screening Kit (Sigma-Aldrich) as per the manufacturer’s guidelines. Donepezil (IC_50_: 40 nM), inducing at least a 50% reduction in enzyme activity, was employed as a positive control.

### 2.12. RNA Isolation and RT-PCR

Following the manufacturer’s guidance, total RNA was extracted (3 × 10^6^ cells) using TRIzol (Invitrogen) and Qiagen TM RNeasy Plus (Qiagen, Valencia, CA, USA) after exposure to TiO_2_ NPs for 24 h at 10 μg/mL. The RNA’s quantity and quality were measured using a NanoDrop (NanoDrop Technologies, Wilmington, DE, USA) and a bioanalyzer (Agilent Technologies, Santa Clara, CA, USA).

PC-12 cells were used to extract total RNA using the RNeasy^®^ Plus Mini kit (QiagenVenlo, Venlo, The Netherlands). In qRT-PCR (QuantStudio3, Applied Biosystems, Foster City, CA, USA), for reverse transcription of RNA, random primers (as specified in Table S1) and SYBR green were used. The target genes’ expression was normalized using glyceraldehyde-3-phosphate dehydrogenase (Gapdh) as a control.

### 2.13. Statistical Analysis

All data for the control group without nanoparticle exposure were reported. Origin Pro 8.0 (OriginLab Corporation, Northampton, MA, USA) examined the nanoparticle characterization data. The in vitro assay data was statistically analyzed using GraphPad Prism 9.4.1 (San Diego, CA, USA) and a two-way analysis of variance (ANOVA) with Tukey’s post-hoc analysis. Each value was investigated using at least three different experiments, and the findings were provided as mean ± SEM.

## 3. Results

### 3.1. Characterization

**Figure 1:**
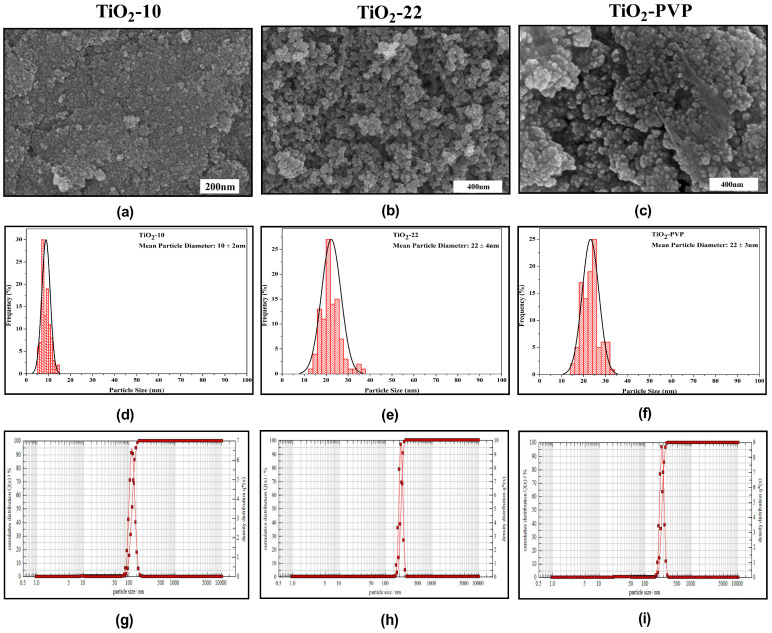
Characterization of synthesized nanoparticles. (**a**–**c**) FE-SEM results depicting morphological properties. (**d**–**f**) Particle size distribution histograms obtained from FE-SEM image analysis. (**g**–**i**) Hydrodynamic diameter determination through dynamic light scattering.

The synthesized NPs were primarily spherical, with few fractions having irregular morphology due to agglomeration, as seen in the FE-SEM images (Figure 1a–c). The mean particle diameter was determined in the histograms (Figure 1d–f). The sample annealed at 500 °C had bigger particles compared to the particle annealed at 400 °C. DLS was used to calculate the hydrodynamic diameter (Figure 1g–i). The results for DLS, particle size, and zeta potential are summarized in Table 1.

**Figure 2:**
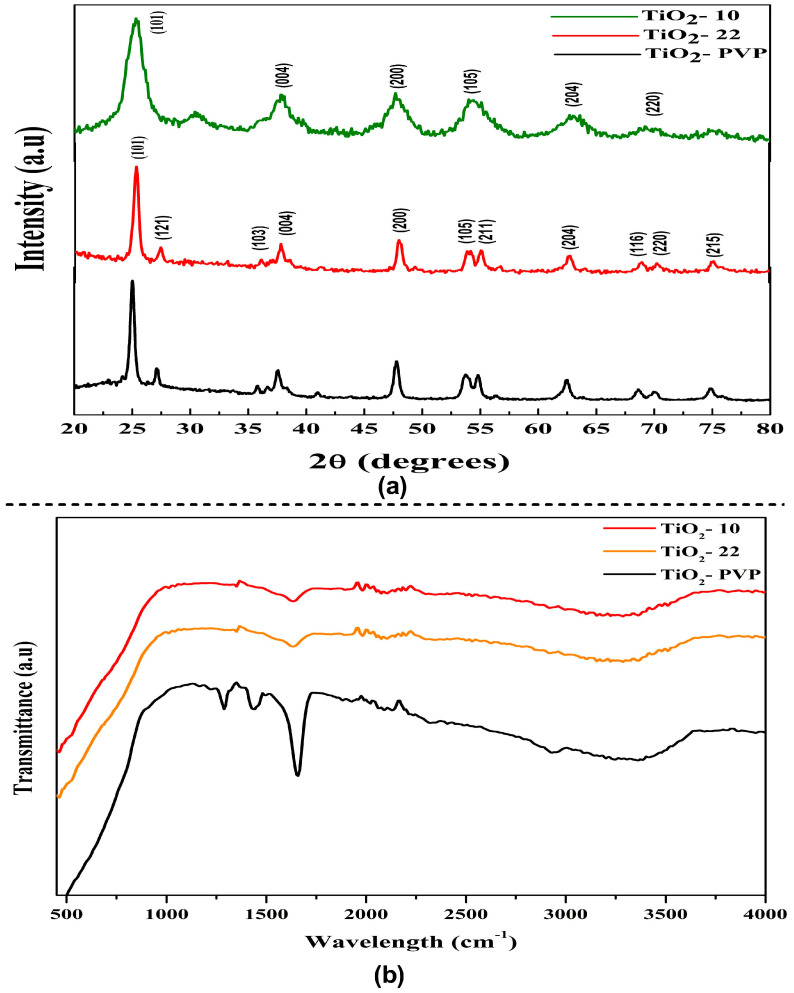
Structural analysis of TiO_2_ NPs. (**a**) X-ray diffraction pattern of TiO_2_ NPs. (**b**) FTIR spectroscopy analysis showing functional groups in synthesized TiO_2_ NPs.

The diffraction peak of the synthesized NPs can be attributed to characteristics peaks that can be attributed to the anatase phase (JCPDS-ICDD card: 21-1272) (Figure 2a). The intense peak diffraction peaks at 25.28, 37.81, 47.99, 53.95, 55, 62.9, 70, and 75 corresponded to the 101, 004, 200, 105, 211, 204, 220, and 215 orientations, respectively. The full width at half maximum (FWHM) of the hkl peaks was determined using the Debye–Scherrer relation to determine the average crystallite size. The estimated crystallite size via XRD was 8 nm, 18 nm, and 20 nm for TiO_2_-10, TiO_2_-22, and TiO_2_-PVP, respectively. The XRD pattern of PVP coated TiO_2_-PVP NPs can be compared with the standard XRD pattern of pure PVP powder (Figure S1). The variance in grain/crystallite was determined to be in good agreement with the FE-SEM results.

As the particle size decreases, the anatase peaks shift towards higher wavenumbers. This phenomenon is known as the quantum confinement effect. The surface of TiO_2_ NPs is heavily hydroxylated. Furthermore, the broadness of the peaks can result from small crystallites. The spectroscopic band is seen at about 3391.72 cm^−1^, and it is explained via the stretching vibrations of the hydroxyl group that are both symmetric and asymmetric (Ti-OH), as seen in the FTIR spectra (Figure 2b). The O-H stretching mode of the hydroxyl group is associated with a broad band between 3600 cm^−1^ and 3000 cm^−1^, which denotes the presence of moisture in the sample. Previous studies have shown that the Ti-O stretching and Ti-O-Ti bridging stretching modes represent the broad band between 1000 cm^−1^ and 500 cm^−1^ [37]. The peak displaying the close relationship between the TiO_2_ NPs and the C=O of PVP was observed at 1660 cm^−1^ (Figure S2), which represents the C=O stretch band for Ti-OH. After synthesizing the PVP-TiO_2_ nanocomposite, this stretching band was re-shifted. At 3400–3500 cm^−1^, the hydroxyl group (-OH) may exhibit symmetrical and asymmetrical stretching vibrations that could be explained by moisture adsorbed on the TiO_2_ surface [38].

**Figure 3:**
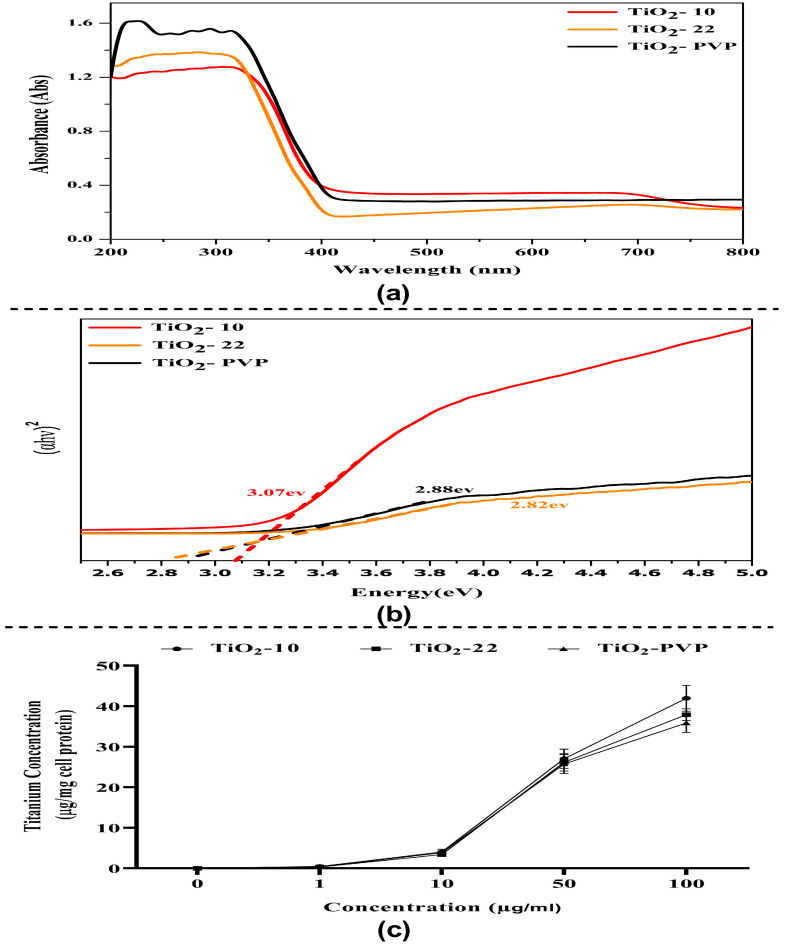
Optical properties and intracellular uptake of TiO_2_ NPs. (**a**) UV–visible absorption spectrum of TiO_2_ NPs in the 200–800 nm range. (**b**) Tauc plot graph for optical energy band gap determination. (**c**) Evaluation of intracellular uptake via total Ti ion quantification via flame atomic absorption spectroscopy.

The absorbance spectrum shows an absorption edge between 270 nm and 320 nm (Figure 3a), which may have been caused by the electron’s photo-excitation while moving from the valence to the conduction band. Many variables may influence a NP’s absorbance, including band gap, oxygen deficiency, and impurity centers. A recent theoretical paradigm by Burello and Worth suggested that the relationship between the cellular redox potential and the metal oxide band gap may shed light on why some substances result in oxidative stress and toxicity [39]. The optical band gap Eg value was obtained from the extrapolation of the linear area of a plot of (αhν)^2^ and energy. The band gap, as measured from the Tauc plot, was found to be 3.07 eV, 2.88 eV, and 2.82 eV for TiO_2_-10, TiO_2_-22, and TiO_2_-PVP, respectively (Figure 3b). This rise in band gap value confirms the difference in the particle size. Additionally, a greater band gap value denotes that the TiO_2_ NPs are more capable of photo-oxidation and photo-reduction.

### 3.2. Total Intercellular Ti Ions

The FAAS analysis suggested a time- and concentration-dependent cellular uptake of TiO_2_ NPs, with no significant difference in the results for all three NPs (Figure 3c and Table S2).

### 3.3. Cell Viability Assay

**Figure 4:**
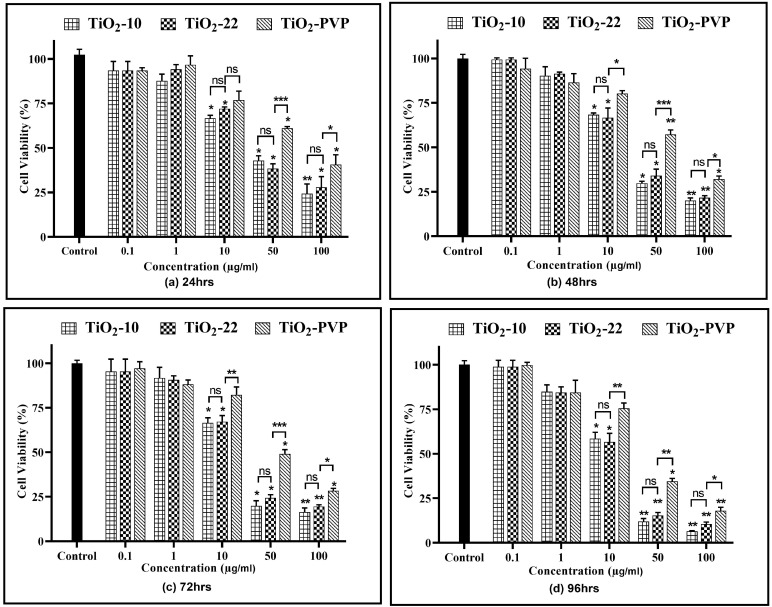
Cell viability assessment via the MTT assay. Viability percentages relative to control cells under the different time points of (**a**) 24 h, (**b**) 48 h, (**c**) 72 h, and (**d**) 96 h. Data are shown as the mean ± SEM (*n* = 3). * *p* < 0.05, ** *p* < 0.01, ^***^ *p* < 0.001, and ns: not significant.

A concentration- and time-dependent reduction in cell viability were observed for all NPs (Figure 4). At 0.1 µg/mL and 1 µg/mL, no cytotoxicity was observed for all three NPs at all-time points compared to the negative control.

For TiO_2_-10, after 24 h of exposure, cellular viability was reduced to below 75% at 10 µg/mL (*p* < 0.05), which further declined slowly with time to 58% (*p* < 0.05) at the end of 96 h. However, at 50 µg/mL, a statistically significant decline was observed at the end of 24 h (42%, *p* < 0.05), which further decreased to 12% (*p* < 0.01) at 96 h. At 100 µg/mL, the cell viability was at 24% (*p* < 0.01) after 24 h and declined to 6% (*p* < 0.01) after 96 h. The results were similar for the TiO_2_-22 NPs, irrespective of the exposure duration compared to the negative control. When the results of both forms of NPs were compared, no statistically significant differences were observed for any concentration and time point. The effect induced via the PVP coating was evident when the outcomes of TiO_2_-22 were paralleled with the coated counterpart (TiO_2_-PVP). The cellular viability at concentrations ≥ 10 µg/mL for TiO_2_-PVP NPs had a significantly lower cytotoxic effect than uncoated TiO_2_-22 NPs of the same size for concentrations ≥ 10 μg/mL at all time points. The IC_50_ was calculated to be 20.57 μg/mL, 18.14 μg/mL, and 28.37 μg/mL after 96 h for TiO_2_-10, TiO_2_-22, and TiO_2_-PVP, respectively.

**Figure 5:**
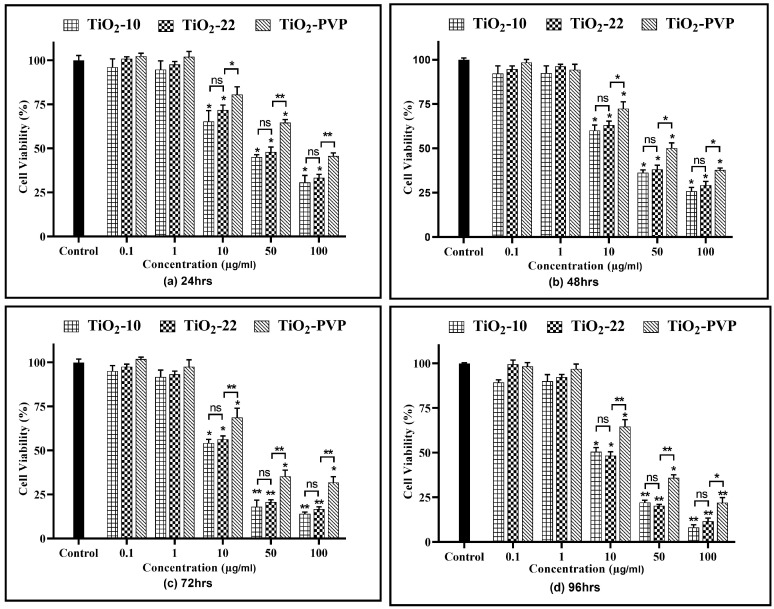
Cell viability assessment via the NRU assay. Viability percentages relative to control cells at the various time points of (**a**) 24 h, (**b**) 48 h, (**c**) 72 h, and (**d**) 96 h. Data are shown as the mean ± SEM (*n* = 3). * *p* < 0.05, ** *p* < 0.01, and ns: not significant.

The results of the NRU assay also indicated a significant concentration- and time-dependent lysosomal activity reduction of active cells (Figure 5). The results suggested a significant (*p* < 0.05) decline in cell viability at 10 µg/mL post 48 h for all NPs as compared to the negative control. At 50 µg/mL for the TiO_2_-10 NPs, there was a significant decline (*p* < 0.05) to below 50% in lysosomal activity at the end of 24 h, which further declined to below 25% (*p* < 0.01) post 96 h. The highest concentration of 100 μg/mL indicated a significant decrease to below 30% (*p* < 0.05) and 10% (*p* < 0.01) after 24 h and 96 h, respectively. When compared to TiO_2_-22, there was no statistically significant difference in the reduction of lysosomal activity at any concentration or duration of exposure between the two differently sized NPs. The PVP coating reduced the cytotoxic effects of NPs, as evident from the significant difference between the uncoated and coated NPs.

### 3.4. Cell Membrane Integrity Assay

**Figure 6:**
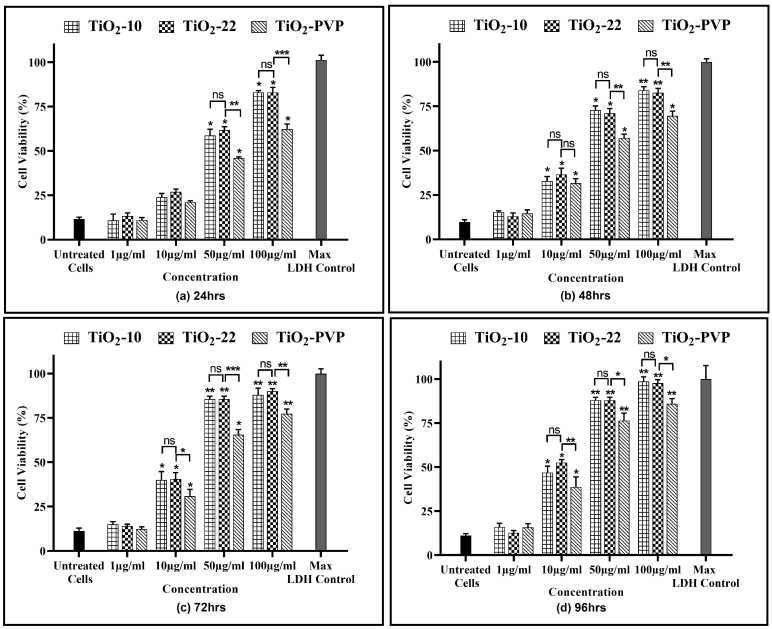
Cell viability assessment via the LDH assay. Viability percentages compared to control cells under the different time intervals of (**a**) 24 h, (**b**) 48 h, (**c**) 72 h, and (**d**) 96 h. Data are shown as the mean ± SEM (*n* = 3). * *p* < 0.05, ** *p* < 0.01, *** *p* < 0.001 and ns: not significant.

The release of the LDH enzyme into extracellular areas signifies cell membrane damage, subsequently leading to cell death. A time- and concentration-dependent LDH leakage was observed for all the NPs, revealing their impact on cell membrane integrity (Figure 6). The results of the LDH assay were in line with the previous MTT and NRU assays, with no significant impact of size on LDH release. However, the impact of the coating was visible, with a significant reduction in LDH enzyme release when compared to the non-coated TiO_2_-22 counterpart.

### 3.5. Mitochondrial Membrane Potential (MMP) Assay

**Figure 7:**
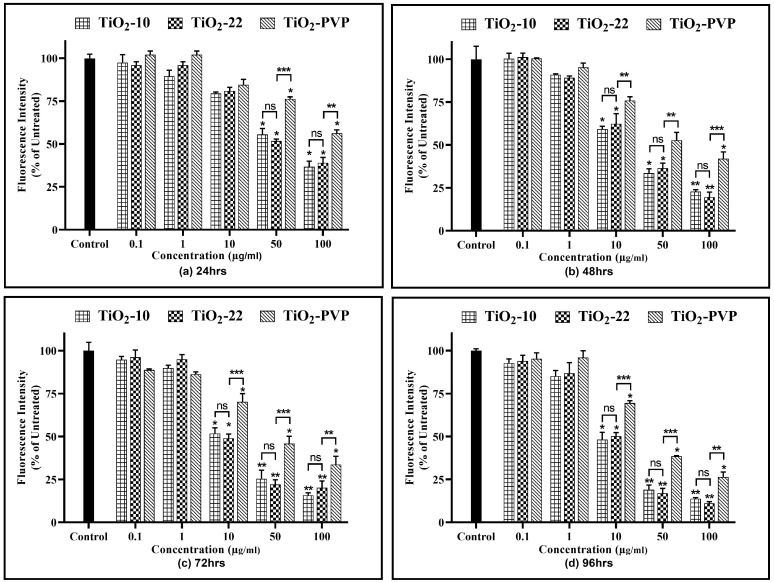
Analysis of the mitochondrial membrane potential (ΔΨm). Changes in the mitochondrial membrane potential relative to control cells at the various time points of (**a**) 24 h, (**b**) 48 h, (**c**) 72 h, and (**d**) 96 h. Data are shown as the mean ± SEM (*n* = 3). * *p* < 0.05, ** *p* < 0.01, *** *p* < 0.001, and ns: not significant.

The depolarization of the mitochondrial membrane, a crucial indicator of mitochondrial health, was assessed via the mitochondrial membrane potential (MMP) as a result of exposure to the TiO_2_ NPs (Figure 7). Compared to the MTT, NRU, and LDH assays, where cytotoxic effects were observed at 10 μg/mL after 24 h, no impact on MMP reduction at 10 μg/mL for all NPs was observed for the same. However, post 48 h, the MMP declined by 46% (*p* < 0.05), 50% (*p* < 0.05), and 59% (*p* < 0.05) post 48 h, 72 and 100 µg/mL, a maximum reduction in MMP to 18% (*p* < 0.01) and 13% (*p* < 0.01) was observed post 96 h. However, comparative analysis for the effect of size on MMP reduction suggested no significant difference in the results at any concentrations or time points. Nevertheless, this decrease in the MMP was slowed down via exposure to TiO_2_-PVP, with significantly higher differences for the same concentration and exposure periods.

### 3.6. Estimation of Reactive Oxygen and Nitrogen Species

**Figure 8:**
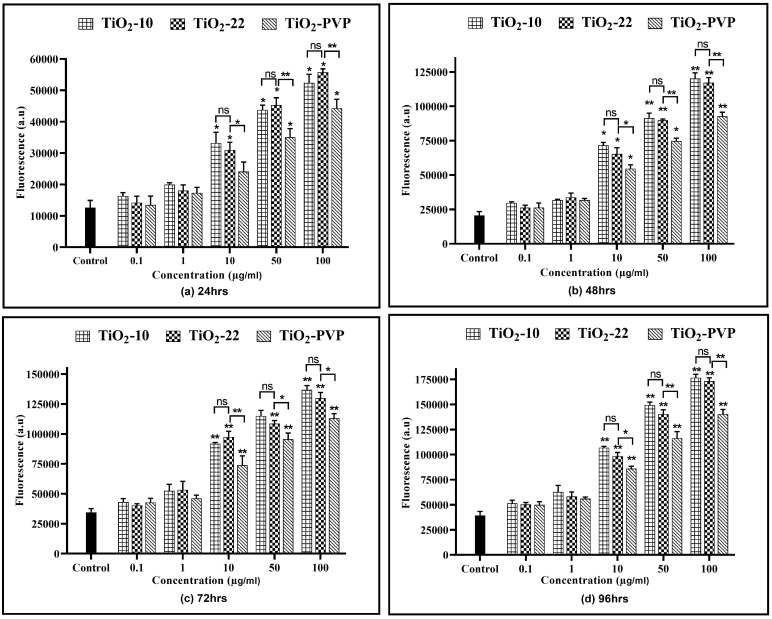
Reactive oxygen species (ROS) analysis. Quantification of ROS levels at the different time intervals of (**a**) 24 h, (**b**) 48 h, (**c**) 72 h, and (**d**) 96 h. Data are shown as the mean ± SEM (*n* = 3). * *p* < 0.05, ** *p* < 0.01, and ns: not significant.

Similar to that of the cell viability assays, at lower concentrations and shorter exposure durations (24 h and 48 h), a significant difference was not observed in the effects produced through all the NPs (Figure 8). At concentrations ≥ 10 µg/mL and extended durations, there was a significant elevation in ROS generation in PC-12 cells post-exposure to TiO_2_ NPs. The ROS levels increased by a minimum of 2.5-fold at 24 h for 10 μg/mL and a maximum by 4.5-fold for 100 μg/mL at 96 h for both the TiO_2_-10 and TiO_2_-22 NPs. However, no significant differences were observed for an increase in ROS at the same concentrations and at any time points for both types of NPs. The comparative analysis of the coated and non-coated NPs suggested a statistically significant difference in the level of ROS, with TiO_2_-PVP generating lower levels of ROS compared to the non-coated TiO_2_-22 NPs.

**Figure 9:**
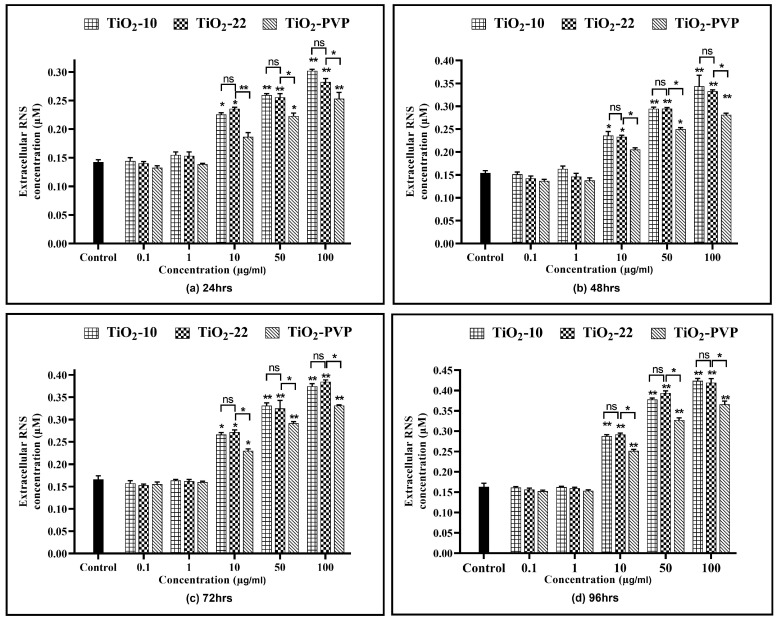
Extracellular reactive nitrogen species (RNS) concentration. Determination of extracellular RNS concentration at the various time points of (**a**) 24 h, (**b**) 48 h, (**c**) 72 h, and (**d**) 96 h. Data are shown as the mean ± SEM (*n* = 3). * *p* < 0.05, ** *p* < 0.01, and ns: not significant.

All three NPs showed a time- and concentration-dependent rise in the RNS levels (Figure 9), except at 0.1 µg/mL and 1 µg/mL. TiO_2_-10 NP exposure indicated a significant 1.52-fold (*p* < 0.05) elevation in RNS levels at all exposure durations for 10 µg/mL compared to the negative control. At 100 µg/mL, there was a significant 2-fold increase in RNS levels at all four time points compared to the negative control. Similar results were also obtained with the TiO_2_-22 NPs. A comparative analysis for the effect of size on RNS levels suggested no significant difference in the levels of RNS when compared to the smaller TiO_2_-10 NPs.

Similar to the ROS assay, the comparative analysis of the coated and non-coated NPs indicated a significant difference in the level of RNS at concentrations ≥ 10 µg/mL at all durations, with TiO_2_-PVP generating lower levels of RNS.

### 3.7. IL-6 and TNF-α Levels via ELISA

**Figure 10:**
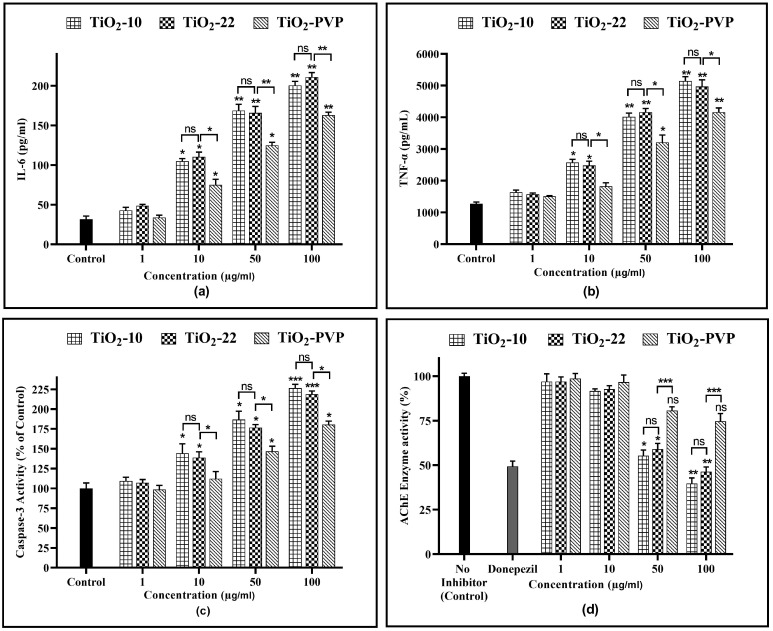
(**a**) IL-6 level determination via ELISA after 24 h. (**b**) TNF-α level determination via ELISA after 24 h. (**c**) Analysis of caspase-3 activity after 24 h. (**d**) AChE enzyme activity inhibition after 24 h. Data are shown as the mean ± SEM (*n* = 3). * *p* < 0.05, ** *p* < 0.01, *** *p* < 0.001, and ns: not significant.

IL-6, a cytokine with multiple functions, is significant in various areas, such as host protection, rapid response reactions, immune responses, nerve cell operations, and blood formation. The results suggest that TiO_2_-NPs are highly effective at stimulating the production of cytokines (Figure 10a). A significantly concentration-dependent increment in IL-6 levels was observed. For the TiO_2_-10 NPs, a 3.35-fold, 5.45-fold, and 6.45-fold increase was observed for 10 µg/mL (*p* < 0.05), 50 µg/mL (*p* < 0.01), and 100 µg/mL (*p* < 0.01), respectively, when compared to the negative control post 24 h of exposure. For the TiO_2_-22 NPs, the IL-6 levels increased by 3.54-fold (*p* < 0.05), 5.32-fold (*p* < 0.01), and 6.8-fold (*p* < 0.01) for the same concentrations, with no significant impact of size. Significant differences in IL-6 levels were seen when coated and uncoated NPs were compared for 10 µg/mL, 50 µg/mL, and 100 µg/mL with a 2.41-fold (*p* < 0.05), 4-fold (*p* < 0.01), and 5.25-fold (*p* < 0.01) increase observed, respectively, for TiO_2_-PVP, which was significantly less than that achieved with the TiO_2_-22 NPs.

The exposure of TiO_2_-NPs to PC-12 cells resulted in similar results as that of the IL-6 assay for the TNF-α levels (Figure 10b). For the TiO_2_-10 NPs, a significant 2-fold (*p* < 0.05) increase was seen at 10 µg/mL, which further increased by 3.15-fold and 4.03-fold for 50 µg/mL (*p* < 0.01) and 100 µg/mL (*p* < 0.01), respectively, when compared to the negative control. For the TiO_2_-22 NPs, the treatment led to a 1.76-fold (*p* < 0.05), 3.1-fold (*p* < 0.01), and 4.8-fold (*p* < 0.01) increase for 10 µg/mL, 50 µg/mL, and 100 µg/mL compared to the negative control, with no significant difference compared to the TiO_2_-10 NPs. The elevation of TNF-α levels was less for coated TiO_2_-PVP NPs than TiO_2_-22 NPs, with statistically significant differences at all concentrations ≥10 μg/mL.

### 3.8. Caspase-3 Activity via ELISA

The amount of caspase-3 activity in the sample was proportional to the strength of the fluorescence or colorimetric signal produced via the assay. When compared to the negative control, TiO_2_-10 NP treatment led to a 1.44-fold increase for 10 µg/mL (*p* < 0.05), which further elevated to 1.86-fold (*p* < 0.05) for 50 µg/mL, and 2.3-fold (*p* < 0.001) for 100 µg/mL. The bigger counterpart, TiO_2_-22, indicated 1.38-fold (*p* < 0.05), 1.76-fold (*p* < 0.05), and 2.18-fold (*p* < 0.001) for 10 µg/mL, 50 µg/mL, and 100 μg/mL, respectively. The elevation of caspase-3 activity following exposure to both TiO_2_-10 and TiO_2_-22 NPs did not differ significantly from one another.

Although substantially less than the uncoated equivalent, caspase-3 activity significantly increased after exposure to TiO_2_-PVP NPs also by 1.46-fold (*p* < 0.05) and 1.8-fold (*p* < 0.05) for 50 µg/mL and 100 μg/mL, respectively.

### 3.9. AChE Activity Inhibition Assay

The AChE activity inhibition results were concentration dependent (Figure 10d). There was no significant impact on enzyme activity at 10 μg/mL for all three NPs. However, at 50 μg/mL, the enzyme activity reduced to 55% (*p* < 0.05) and 59% (*p* < 0.05) with the TiO_2_-10 and TiO_2_-22 NPs, respectively, further declining to 39% (*p* < 0.01), and 46% (*p* < 0.01) at 100 μg/mL. Compared to the uncoated NPs, the coated TiO_2_-PVP had shown a decline in activity at 50 µg/mL and 100 μg/mL, but the results were insignificant.

### 3.10. TiO_2_ NP Effect on Dopaminergic Gene Expression

**Figure 11:**
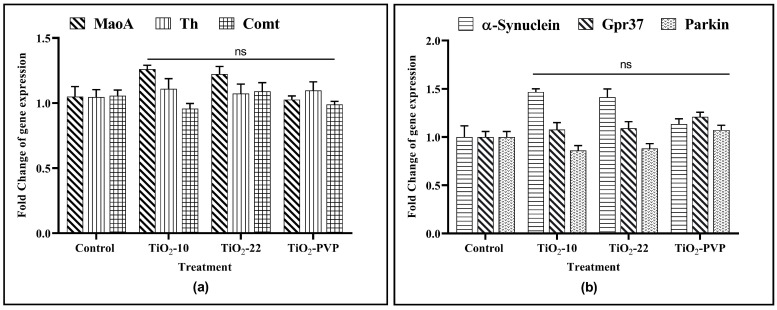
Gene expression analysis upon exposure to TiO_2_ NPs. (**a**) Gene expression changes of MaoA, Th, and Comt. (**b**) Gene expression changes of α-synuclein, Gpr37, and parkin. Data are shown as the mean ± SEM (*n* = 3) with reference to the control. ns: not significant.

The 24 h treatment of PC-12 cells with TiO_2_ NPs at 10 μg/mL showed no significant impact on the gene expression of any of the six genes associated with dopamine metabolism and Parkinson’s etiology (Figure 11).

## 4. Discussion

As mentioned previously, the NPs synthesized for the current study were within the size limit (≤100 nm), which has a high possibility of permeating across the BBB. The current study’s concentration range of 0.1–100 μg/mL was selected based on the literature. However, it is important to emphasize the difficulty of figuring out the average release of particles owing to tribocorrosion from Ti implants, food additives, and packaging materials due to multiple parameters, such as the implant’s composition, type, surface area, and duration of exposure.

Ti does not have a physiological role in the human body. As a result, detecting Ti residues in the body is categorically regarded as a contaminant. Previous in vivo studies on bioaccumulation have suggested that only a tiny fraction of TiO_2_ NPs from the dose administered can reach the CNS by crossing the BBB and that this is enough to induce oxidative stress, despite not being in the detectable range [40,41]. This distinction is critical for comprehending the relevance of Ti exposure and its potential health consequences, as the NPs can integrate into cellular membranes via endocytosis and subsequent fusion with lysosomes, eventually causing adverse biological responses in neural cells.

The current study started with assessing the cellular viability of the neural PC-12 cells via the MTT, NRU, and LDH assays. The results suggested a time- and concentration-dependent decrease in cell viability with no significant size impact in all three assays. However, the PVP coating was able to significantly reduce the level of cytotoxicity. This was evidenced with calculated IC_50_ values of 20.57 µg/mL, 18.14 µg/mL, and 28.37 μg/mL at the end of 96 h for TiO_2_-10, TiO_2_-22, and TiO_2_-PVP, respectively. The results also showed an IC_50_ of 34.51 µg/mL and 36.23 μg/mL at 48 h of exposure for TiO_2_-10 and TiO_2_-22. This was in line with one of the studies on rat primary cultured hippocampal neurons, which suggested IC_50_ values at 32.35 μg/mL after 48 h of exposure [42]. Further, the FAAS analysis indicated no significant difference in the concentration of NPs by the PC-12 cells.

Cytotoxicity has been reported at a much lower concentration than the concentration of 10 μg/mL observed in the current research. However, a study on the primary culture of olfactory bulb neurons suggested a significant reduction in viability at 5 μg/mL. This concentration was further reduced to 1.25 and 2.5 μg/mL in a few other studies with significant inhibition of neurite development in cultured rat primary hippocampal neurons and primary cortical neuron cultures upon exposure ranging from 24 h to 8 days [43,44,45]. This low-concentration cytotoxicity may be due to the high sensitivity of primary cultured neurons compared to PC-12 cells.

On the other hand, multiple previous research studies have reported contradicting results. A study on PC-12 cells reported a maximum decline of 20–40% after 24 h of exposure to 20–50 nm TiO_2_ NPs, even at their highest concentration of 50 µg/mL and 125 μg/mL, which was much lower than the results obtained in the current study [18]. Another study reported a complete absence of cytotoxicity even at 100 μg/mL after 4 days of exposure in the PC-12 cells [46]. Similar results were also reported in other neural cells, such as SHSY-5Y cells, where no cytotoxicity was reported at any concentration (0–150 μg/mL) post 3 h, 6 h, and 24 h exposure to 25 nm TiO_2_ NPs through both the MTT and NRU assays, despite reporting a concentration-dependent increase in apoptosis [47]. One of the primary reasons for this may be the absorbance of almost 70% of incident UV by TiO_2,_ as reported previously [48]. However, in the current study, not only was the absorbance of incident UV via NPs considered while evaluating the MTT results, but we also determined cytotoxicity via other assays, such as the NRU and LDH assays. The outcomes from all three different cytotoxicity assays aligned and suggested time- and concentration-dependent cytotoxicity.

In conjunction with the MTT assay, we also studied the alterations in the MMP to understand the integrity and functionality of the mitochondrial membrane, which is critical for cellular energy production. TiO_2_ NPs may deplete the resources required to produce high-energy phosphate. The results revealed a considerable concentration-dependent decline in the membrane potential with no size impact. A significant difference in the MMP reduction was observed between the TiO_2_-22 and TiO_2_-PVP NPs, suggesting a higher disturbance in the electron transport chain across the mitochondria via uncoated NPs compared to their coated counterparts.

A decline in the MMP could indicate electron transport chain (ETC) dysfunction, which interferes with the chain’s ability to conduct electrons, as this flow is disrupted. This dysfunction leads to the leakage of electrons from the ETC, which can interact with molecular oxygen, consequently leading to the incomplete reduction of oxygen molecules [49]. Within the mitochondria, superoxide radicals are produced due to incomplete oxygen molecule reduction, including ROS, which can harm cellular components, such as lipids, proteins, and DNA. These elevated ROS levels can cause cellular stress responses and, in severe cases, apoptosis (programmed cell death), adversely affecting cellular health and function. The findings of this research demonstrated a concentration- and time-dependent elevation of ROS and RNS levels for all three NPs. The prolonged exposure of TiO_2_ NPs to PC-12 cells at concentrations ≥ 10 μg/mL indicated a 2-fold increase in oxidative species levels. The results were in line with a previous study on human (astrocytes-like) D384 cells, which reported a trigger in ROS generation at a much lower concentration of 1.5 μg/mL [50].

While the cell’s endogenous antioxidant enzyme system is likely initiated in response to counteract the heightened levels of ROS, it is plausible that the ensuing cellular response falls short in terms of effectiveness. This was seen in one of the studies on rat alveolar macrophages exposed to TiO_2_ NPs, where, despite increased antioxidant enzyme levels, lipid peroxidation and hydrogen peroxide generation remained elevated [51]. This suggested that TiO_2_ NPs might cause oxidative stress, which would then trigger the induction of antioxidant enzymes as a means of self-defense in the cells, which may not be adequate to counteract the harmful effects of TiO_2_ NPs. One of the reasons may be attributed to an imbalance in antioxidant enzyme production triggered via the TiO_2_ NPs, as observed in rat C6 and human U373 glial cells in another study. This study reported a maximal increase in ROS at 6 h, which then declined at 24 h in both cells. In the same study, the outcomes for the expression of antioxidant genes suggested similar results, with an increase in GPx, SOD2, and catalase expression levels at short exposure (at 6 h and 24 h) but a decrease at more prolonged exposure (at 48 h and 72 h) [52].

Excessive ROS generation may also trigger inflammatory signals in addition to oxidative stress and apoptosis. IL-6 and TNF-α are classified as pro-inflammatory cytokines that neurons and other cells can discharge in reaction to stimuli such as injury, infection, or stress. CNS immune responses are associated with a complex role in neuroinflammation [53]. Chronic neuroinflammation can damage neurons and disrupt communication, leading to cognitive impairment, motor deficits, and other neurological symptoms, as seen in previous in vitro studies upon exposure to TiO_2_ NPs [53,54,55,56,57]. The current study also observed a significant concentration-dependent (≥10 μg/mL) increase in IL-6 and TNF-α levels post 24 h of exposure in PC-12 cells, raising noteworthy implications, especially in the context of NDs.

The oxidative stress triggered by TiO_2_ NPs corresponds to a toxic mechanism in the CNS. Both mitochondrial dysfunction and oxidative stress trigger apoptotic caspases [58]. Elevated ROS levels and RNS activate apoptotic signaling pathways, activating caspases via proteolytic cleavage. On the other hand, mitochondrial dysfunction triggers the release of cytochrome c in the cytoplasm, forming a complex known as the apoptosome, leading to the activation of caspase-3 [59]. The combined effect of both phenomena was observed in the current study with a minimum of 1.3-fold at 10 μg/mL and a maximum 2-fold rise at 100 μg/mL in caspase-3 activity upon exposure to TiO_2_-22 and TiO_2_-10 NPs. The coated TiO_2_-PVP, however, only exhibited a significant effect at concentrations ≥ 50 μg/mL. The results aligned with a previous study on mouse hippocampal neuron HT22 cells, which reported increased expression of caspase-3 and Bax, and decreased expression of Bcl-2, suggesting a prominent role of oxidative stress in TiO_2_ NP-induced apoptosis [60].

Multiple studies have also shown that NPs can engage with acetylcholinesterase (AChE), which would disrupt its normal enzymatic function. By physically binding to the AChE active/binding sites, these NPs can alter the enzyme’s structure [61]. Due to this fact, it is thereby possible that the enzyme’s ability to bind to its substrates and carry out its activity would both drop. Due to the imbalanced acetylcholine levels that result from these interactions, cholinergic neurotransmission is decreased, which can significantly affect brain functions [62,63]. The current study suggested a concentration-dependent decrease in AChE activity but with no significant difference between the TiO_2_-10 and TiO_2_-22 NPs. However, compared to the uncoated NPs, the PVP coating significantly decreased the exposed surface area, which may have contributed to the PVP coating’s ability to diminish the harmful effect of TiO_2_ NPs. These findings were similar to multiple studies that suggested an inhibition in acetylcholinesterase enzyme activity apart from an increase in oxidative stress and apoptosis upon exposure to TiO_2_ NPs [61,62]. In addition, AChE is also vulnerable to deactivation due to oxidative stress triggered via reactive oxygen species (ROS) [64,65], which goes beyond their conventional surface adherence to NPs. However, contrary to this, a study on CD-1(ICR) female mice reported increased AChE activity after intranasal instillation of TiO_2_ NPs, despite indicating an increase in oxidative stress [66].

The relationship between the inhibition of the AChE enzyme and neurological diseases has been the subject of extensive scientific research. To better understand it, the complex interactions between the TiO_2_ NPs and dopaminergic systems were studied in the current research. For this, three genes associated with dopamine metabolism and three genes associated with the etiology of NDs were studied for the changes in their gene expression upon interaction with the TiO_2_ NPs to determine the relationship between the pathophysiology of neurodegeneration in Parkinson’s disease (PD) and the metal NP-induced neurotoxicity. While investigating the impact of TiO_2_ NPs, an increase in α-synuclein expression was observed for TiO_2_-10 and TiO_2_-22, but it was found to be insignificant. Apart from this, no change in expression for any other genes related to dopamine metabolism or ND etiology for any NP exposure at 10 μg/mL was observed. However, one of the previous studies on PC-12 cells did report an increase in α-synuclein expression but at 50 μg/mL, which was five times more than the concentration observed in the current study [67].

Consequently, the outcomes of this study indicate that TiO_2_ NP-induced cytotoxicity is due to the physiochemical properties of the NPs themselves. A tendency for causing apoptosis and triggering inflammatory cascades in brain cell populations comes to the fore when these NPs scale down in size, converging toward diameters around zero nanometers. It is interesting to note that these results remained consistent, regardless of the size of the NPs. However, the presence of surface coatings for similar-sized particles limited their ability to induce detrimental cellular reactions in neural PC-12 cells. As the findings revealed that these NPs can cause apoptosis and inflammatory responses through oxidative stress, it emphasizes the need for careful consideration of their potential effects in neurobiology. Accordingly, the research outcomes guide future research, influencing the development of safer nanomaterial uses and improving our understanding of their complex interactions inside cellular systems.

## 5. Conclusions

According to the report on regulatory reviews on nanomaterials, since there is no universally applicable method for identifying the hazards of NPs, an individualized, case-by-case approach is still required for their risk assessment [68]. Specifically, the toxicological evaluation must be performed based on the properties and characteristics of the material in question, using selected criteria, methods, and strategies. Standardized tests for the assessment of NP safety do not exist. Ideally, the preliminary screening of nanotoxicity occurs in vitro, although it is necessary to identify reliable models that can more accurately predict and mimic the in vivo environment. Developing trustworthy models with high predictive capacity for nanotoxicity testing is essential.

In conclusion, oxidative stress, apoptosis, and inflammatory responses were induced using the three distinct TiO_2_ NPs. Furthermore, our findings suggest that the differences between the NPs, mainly the toxicity induced, may not differ considerably when the size approaches the zero scale (nm), but that the coating might play a vital role in such scenarios.

## Figures and Tables

**Table 1:** The results for size analysis, hydrodynamic diameter, and zeta potential of synthesized TiO_2_ NPs.

Nanoparticle	Size via FE-SEM (nm)	Hydrodynamic Diameter (nm)	Zeta Potential (mV)
TiO_2_-10	10 ± 2	164.74	−22.32
TiO_2_-22	22 ± 4	262.88	−26.17
TiO_2_-PVP	22 ± 3	293.24	−27.58

## Data Availability

Data will be made available on request.

## Supplementary Materials

The following supporting information can be downloaded at: https://www.mdpi.com/article/10.3390/jox13040043/s1.
