# Supplementary material for: Comprehensive Analysis of Titanium Oxide Nanoparticle Size and Surface Properties on Neuronal PC-12 Cells: Unraveling Cytotoxicity, Dopaminergic Gene Expression, and Acetylcholinesterase Inhibition
Source: J Xenobiot. 2023 Nov 7;13(4):662–84. doi: 10.3390/jox13040043 (PMC10660528; doi:10.3390/jox13040043)

SUPPLEMENTARY DATA

Comprehensive Analysis of Titanium Oxide Nanoparticle Size and Surface Properties on Neuronal PC-12 Cells: Unraveling Cytotoxicity, Dopaminergic Gene Expression, and Acetylcholinesterase Inhibition

1. Figure S1. X-Ray diffraction pattern of pure PVP powder.

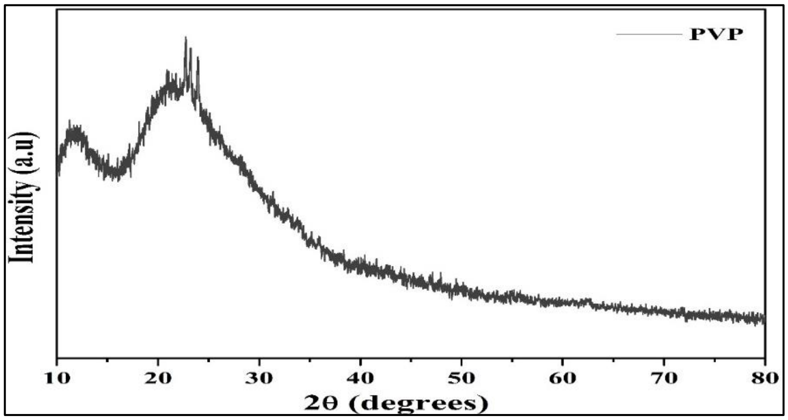

2. Figure S2. FTIR spectra of pure PVP powder.

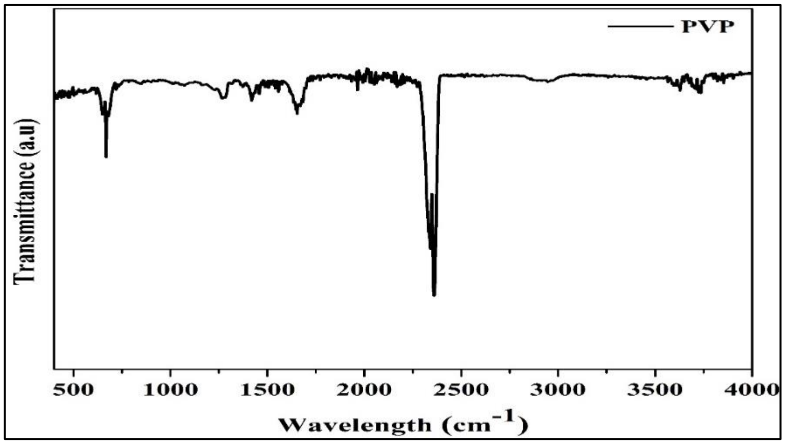

3. Supplementary Table S1. List of primers for gene expression studies.

Primer Name	Primer Sequence 5' --> 3'
Tyrosine hydroxylase (Th)- 1F	CCTTCCAGTACAAGCACGGT
Tyrosine hydroxylase (Th)- 1R	TGGGTAGCATAGAGGCCCTT
Monoamine oxidase A (Maoa)- 1F	GAACCCGAGTCCAAGGATGT
Monoamine oxidase A (Maoa)- 1R	ATGGCCCAAACCATAGGCTG
Catechol-O-methyltransferase (Comt)- 1F	GTTAAAACCCGTGTCTGCGG
Catechol-O-methyltransferase (Comt)- 1R	CATGATTTGGCCTTTCGCGT
α-synuclein 1 F	GACCAGATGGGCAAGGGTGA
α-synuclein 1R	CATAAGCCTCACTGCTAGGGT
GPCR-37 -1F	CAACCTCGCTTTCTGGGACT
GPCR-37 -1R	ACCCAGAGAAGCTACCTCGATA
Parkin -1F	CGGGACTGCAAGGAAGCATA

Parkin-1R	CCGGTATGCCTGAGAAGTCG

4. Supplementary Table S2: Data for validation of TiO_2 NP's concentration by Flame Atomic Absorption Spectroscopic Assay.

(a) Method settings.

**Replicates**	**3 Sample Uptake**
Time (s)	40
Calibration Correlation Coefficient Limit	0.999
fast pump	On
Pump Speed (rpm)	15
Blank Subtraction	On
Sample Introduction	Autosampler
Stabilization Time (s)	30
Element	Ti
Label Wavelength (nm)	498.73
Calibration Fit	Linear Weighted
Nebulizer Pressure (kPa)	240

(b) Calibration Curve.

**Sample**	**Concentration ((µg/ml)**	**Intensity**
Blank	0	0.00
Standard 1	0.5	108.30
Standard 2	1	233.58
Standard 3	5	1080.98
Standard 4	10	2316.57
Standard 5	50	1160.29
Standard 6	100	23550.03

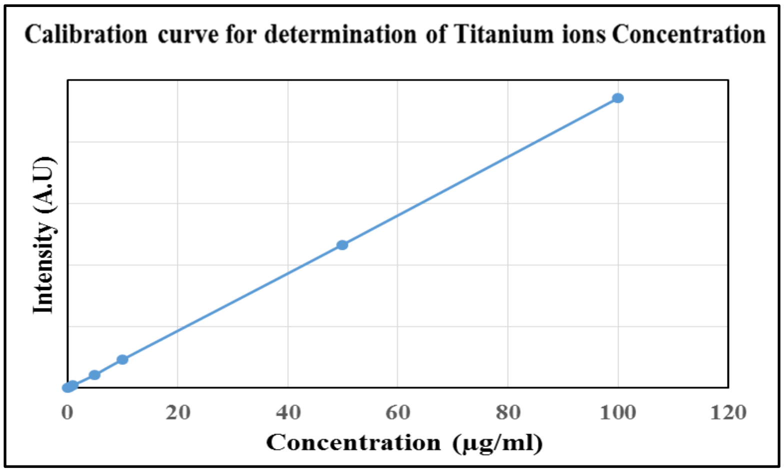

Supplement: Supplementary file 1 [file jox-13-00043-s001.zip › jox-2617645-supplementary.pdf]
